# Supplementary material for: Prognostic value of preoperative circulating tumor cells counts in patients with UICC stage I-IV colorectal cancer
Source: PLoS One. 2021 Jun 10;16(6):e0252897. doi: 10.1371/journal.pone.0252897 (PMC8191913; doi:10.1371/journal.pone.0252897)
Supplement: Attachment — Submitted filename: Response to Reviewers.doc [file pone.0252897.s002.doc]

Dominique Heymann, Ph.D.
Academic Editor
Public Library of Science

*PLOS ONE*

Thaer S. A. Abdalla

Department of General Surgery

University Medical Center Hamburg-Eppendorf

Hamburg, Germany

20.05.2021, Hamburg

Dear Prof. Heymann,

Thank you very much for giving us the opportunity to resubmit our manuscript in your highly respected journal.

In the following you will find point by point answers with regard to the editorial comments as well as the comments by the reviewers. Each table was revised in accordance to the journal’s guidelines. All changes are clearly marked in the revised manuscript.

We would greatly appreciate to publish our work in the “*PLOS ONE*” and ask for a revised review process.

Sincerely yours,

Thaer S. A. Abdalla

**Editorial comments:**

1. ***Comment and discuss the size and shape of CTCs dectected (ie. presence of clusters)***

Thank you for your kind remarks. Keratin-positive nucleated cells of round or oval shape with a diameter of at least 4 µm and CD45 negativity were considered CTCs. Only in a minority of cases CTC clusters with 2 or 3 CTCs were detected.

2. ***Comment and discuss* the limit of the technique used based on EpCAM marker**

As requested, the limits of the anti-EpCAM methods have been discussed in the lines 174-185 pages 9-10.

3. ***Changes in the reference list***

The list of references has been updated in order to answer the requested changes. The references 27-30 and 32-34 have been recently added.

**Reviewer 1:**

First of all we would like to give our thanks to the Reviewer 1 on his thoughtful comments and suggestions.

1. ***The authors should state in their discussion that this is not the first study to investigate CTC in non-metastatic colorectal cancer and discuss related work in more detail. E.g. the article "Circulating tumour cells and outcome in non-metastatic colorectal cancer: a prospective study. Br J Cancer. 2015 Apr 14;112(8):1306-13. doi: 10.1038/bjc.2015.88. " should be cited and discussed, as this was the first trial to investigate non-metastatic CTC count in CRC with the Cell search system.***

Thank you for this remark. We absolutely agree that there are previous studies to investigate CTCs in non metastastic colorectal cancer like the one by Bork et al. Thus, we added a revised description on page 10 of the manuscript.

In our work we used the CellSearch® system for identifying CTCs in all stages of CRC scheduled for resection with curative intent and compared their detection preoperatively and within 96 hours postoperatively to the OS and PFS.
